# Past, present, and future of COVID-19: a review

**DOI:** 10.1590/1414-431X202010475

**Published:** 2020-07-24

**Authors:** C.M. Romano, A. Chebabo, J.E. Levi

**Affiliations:** 1 Hospital das Clinicas HCFMUSP (LIM52), Faculdade de Medicina, Universidade de São Paulo, São Paulo, SP, Brasil; 2 Hospital Universitário Clementino Fraga Filho, Universidade Federal do Rio de Janeiro, Rio de Janeiro, RJ, Brasil; 3 Laboratórios Dasa, São Paulo, SP, Brasil; 4 Laboratório de Virologia, Instituto de Medicina Tropical, Universidade de São Paulo, São Paulo, SP, Brasil

**Keywords:** SARS-CoV-2, COVID-19, RT-PCR, Diagnosis, Origin, Treatment

## Abstract

SARS-CoV-2 has recently emerged, becoming a global threat, affecting directly all human beings owing to its morbidity and mortality and indirectly, due to the enormous economic and psychological impact produced by social isolation, the most effective measure so far, but unsustainable for a long period. The scientific effort to understand and control SARS-CoV-2 transmission and clinical impact has been huge, and important achievements are highlighted in this review. Diagnosis is central and is the first step in recognizing and fighting any infectious agent. Instrumental to that is the quality of the data, relying on serological and molecular surveys in addition to trustworthy clinical records. However, the fast spread of a virus adapted for human-to-human respiratory transmission raised a demand for millions of molecular tests that are simply not available. Several candidate drugs are under evaluation in clinical trials. Those with an already recognized safety profile are more auspicious, since, if proven effective, can cut several steps of production and phase 2 and 3 trials. More than one hundred vaccine prototypes are in different stages of development, however, safety and efficacy evaluations cannot be obviated, implicating, most optimistically, in at least months for us to have an effective immunization, the definite measure to allow a safe return to the pre-pandemic lifestyle. Science has never been more necessary and present in daily life. Relying on the best of human wit is the only way out to this pandemic, saving as many lives as possible.

## Introduction

It is quite a challenge to write a review on the most recently emergent coronavirus (SARS-CoV-2) and the disease caused by it, named COVID-19. In a few months, thousands of papers were produced by all scientific branches, impossible to be followed, properly rated, and summarized. We will focus here on our areas of expertise: molecular epidemiology, diagnostics, and clinical management, briefly touching other aspects.

Sadly, we have to admit that the global community, including scientists working closer to the problem, such as virologists, infectologists, epidemiologists, vaccinologists, public health authorities, and the pharmaceutical industry, collectively failed in avoiding this pandemic to occur. It was not unpredictable; in fact, it was anticipated by many (1,2), and we had at least two “rehearsals”, SARS-CoV in 2002/2003 and MERS-CoV in 2012, that provided enough evidence of the risk and harm of coronaviruses jumping from animals to humans (3). There was time enough for the development of a pancoronavirus vaccine and treatments. This mistake is now causing thousands of lives to be taken directly by viral action and indirectly by the economic deprivation that follows.

As we write this manuscript, Brazil presents the 2nd highest absolute number of cases, approximately two million, and deaths, now reaching 70,000 (4). Due to its large population and territory, intense flow of international passengers and immigrants from all over the world, and many densely populated urban centers, the country has a fertile environment for emerging agents. The Zika virus crisis in 2014 is an example of an arthropod-borne disease that in Brazil has shown a previously unknown feature of a flavivirus human infection: the ability to cause fetal growth anomalies (5). More recently, we were at a perilous limit for the resurgence of urban Yellow Fever, avoided by a massive immunization effort (6), while Dengue viruses keep causing thousands of infections and hundreds of deaths every year (7). Even with all these warnings, a public health strategy to prevent or minimize the risk of another emergent infectious agent is not in place. We lack a robust surveillance system with advanced testing technologies able to rapidly identify such agents and react to them. It is certainly very expensive but undoubtedly cost-effective. This should be one painful lesson we take as a country but that could lead the world to become more strongly connected.

## SARS-CoV-2 origin and spread

### Origin

The earliest report of a cluster of an atypical pneumonia, later named SARS-CoV-2, dates from late December 2019, and it was linked to people that visited the Huanan seafood and wildlife market in Wuhan city in Hubei province, China (8,9). Analyses of nucleotide sequences of complete SARS-CoV-2 genomes led to the estimation that the virus might have emerged as early as October 2019, therefore a couple months before its first detection (10).

SARS-CoV-2 belongs to the subgenus Sarbecovirus of the genus Betacoronavirus, and phylogenetic analysis demonstrated that it is more related to two bat-SARS-like coronaviruses (88% identity at nucleotide level) than to the human SARS-CoV and MERS-CoV (79 and 50%, respectively). Further analysis has revealed 95.4% identity between the SARS-CoV-2 to a coronavirus found in pangolins (Pangolin-CoV), suggesting a possible intermediate host (11,12).

Although no animal coronavirus sampled so far is sufficiently similar to SARS-CoV-2 to implicate it as direct source to the human virus, the most plausible hypothesis tells us that an animal hosted SARS-CoV-2-like jumped into humans, and during the first stages of human-to-human transmission, it acquired very particular genetic features. The ‘humanized' virus then became highly infectious and able to efficiently spread among humans.

While all evidence points to a zoonotic origin of the SARS-CoV-2, there were conspiracy theories claiming that it could have been deliberately created. Some genetic particularities, like the presence of specific mutations in the receptor-binding domain (RBD) of the spike protein, provide definite evidence that the new coronavirus is not a product of human manipulation. Analysis of this region among human and animal SARS-CoV-2-related viruses revealed that the RBD of SARS-CoV-2 evolved for binding to human ACE2 receptor with an efficient interaction, distinct to those predicted through structural and functional analysis (13,14).

### Phylogenetic analysis

Just few months after the first genome description in January 2020, thousands of global SARS-CoV-2 partial and complete genome sequences were generated by several groups, allowing epidemiological and all sorts of genetic and phylogenetic analyses. Until the writing of this text, 49,607 SARS-CoV-2 partial and complete genomes have been sequenced worldwide and made available at repositories such as GISAID (https://www.epicov.org). Although the sequencing efforts vary considerably among the countries and regions, initial analysis demonstrated some structured geographical distribution, which becomes weaker as the epidemic progresses. Some organizations and institutions have made efforts to sequence rapidly and on a large scale, as the COVID-19 Genomics UK (COG-UK) Consortium, from the United Kingdom, and the SPHERE, from the Centers for Disease Control and Prevention (CDC, USA). In Brazil, nearly 500 complete genomes have been sequenced and made available at a public database (https://www.gisaid.org). Different efforts and consortia for this purpose are ongoing and include state and federal institutions and universities including Oswaldo Cruz Foundation, University of São Paulo, São Paulo State University, Campinas State University, and the Federal Universities of Minas Gerais, Tocantins, Brasilia, and Rio de Janeiro, among others.

Geographical and phylodynamic inferences are possible as more representative viral genomic data become available. Currently, it is possible to recognize up to ten SARS-CoV-2 lineages (being A1a, A2a, B, and B1 the most widespread). So far, viruses from A2a, A1a, and B lineages were described in Brazil, with more than 90% of sequences belonging to the A2a lineage (NEXTSTRAIN, <nextstrain.org>). In fact, the increase in frequency of the A2a (which carries a mutation D614G in the spike protein) has been explosive, and today it is the most widespread lineage globally. Some speculation exists that this lineage may present better fitness than the original one, or that it is more infectious and adapted to human cell receptors (15,16), but not enough data is available at the moment to confirm or refute any of these theories.

The analysis of the first sequences sampled in Brazil revealed that they were imported mainly from Europe and the USA and the epidemic here is a result of multiple and distinct introductions (17). With few exceptions (18), it has been recognized that the outbreak in nearly all countries is the result of multiple introductions from different places into the region and not from a single source (19–24).

SARS-CoV-2 evolutionary rate was estimated to be of about 8×10^-4^ substitutions per site per year, which is similar to the related SARS-CoV-1 and MERS (25). Comparison analysis among worldwide isolates revealed moderate genetic diversity accumulated at this point of the pandemic. Pairwise comparisons reveal no more than 10 single nucleotide polymorphisms (SNPs) between any two genomes, thus resulting in >99.9% similarity among SARS-CoV-2 genomes. Although some of these SNPs result in non-synonymous mutations, none of them have shown significant shifts in the functional context of the respective proteins or in the transmission rates. Nevertheless, as is the case for any other pathogen, nucleotide mismatches could lead to a lower sensitivity and inclusivity of the molecular diagnostic assays, thereby resulting in false-negative diagnosis (26).

In sum, phylogenetic, clinical, and epidemiological data together offer insights into how the viruses are related and evolve over time. At the moment, data from the SARS-CoV-2 genomes indicate that we still have much to learn about the evolution of this virus and how it spreads.

## Diagnosis

Management of infectious diseases always begin at the identification of the agent. Therapeutic and preventive strategies start by taking into account the kind of microorganism, its transmission routes, and spreading capacity. As pointed out above, SARS-CoV-2 was quickly recognized as the etiological agent by the most modern diagnostic technology available today, metagenomics (27), which consists of the unbiased study of the microbial diversity directly from a clinical or environmental sample by high-throughput DNA sequencing. By excluding DNA from human origin, either *in vitro* or *in silico*, researchers can focus the analysis on non-human sequences, disclosing microbial diversity and allowing the discovery of new agents (28).

By this approach, on January 11, 2020, the full-sequence of the first new coronavirus genome was made publicly available by Chinese scientists. The availability of the first whole genome 29,903 bases RNA sequence made possible the development of real-time PCR protocols, forming the groundwork of SARS-CoV-2 identification in symptomatic patients. From this date on, many RT-PCR protocols were created, targeting different genomic regions. The non-structural proteins seem to accumulate diversity faster than the structural ones, in particular nsp2, 3, and 4, and some insertions and deletions are also common along ORF8. Complete genome analysis suggests that the conserved Orf1b, envelope and spike encoding genes are more conserved, hence better targets for molecular assays, whereas the N gene displays more variability between current isolates (26,29).

As the number of cases grew exponentially, it was realized that the global availability of reagents for RNA extraction and PCR amplification was much inferior to the actual need for the diagnosis of symptomatic subjects and to assist selective social isolation.

Currently there are dozens of PCR kits for SARS-CoV-2 diagnosis in respiratory samples, targeting one, two, or three distinct genomic fragments (see Table 1). Including multiple targets is a strategy to cope with the uncertainty of the mutation rate of each gene, which can lead to false-negatives due to primer mismatches. For instance, the original PCR protocol described by the Charité group from Berlin (30) proposed to screen the samples with an E-gene pan sarbecoviruses (a subgroup of beta-coronaviruses) and to confirm reactive samples with an assay targeting specifically the SARS-CoV-2 RNA dependent RNA polymerase viral gene (RdRP). Later, the RdRP assay was found to lead to false-negatives in some isolates (31,32). In general, many assays use the panSarbeco E gene amplimer in addition to a second target, either in parallel or as a sequential confirmatory approach. The US CDC had initially proposed 3 targets on the nucleocapsid gene (N1-N2-N3) but later withdrew the third one (N3) due to low efficiency (33). Primers and probes spanning the open reading frames 1 and 3 have also been adopted in different combinations. One single target permits higher throughput testing and makes interpretation easier in comparison to two or three targets, which demands more complex interpretation algorithms and increase the number of indeterminates.

**Table 1:** Genomic regions targeted by different RT-PCR systems for SARS-Cov-2 and their reported analytical sensitivity.

Institution/Manufacturer	SARS-CoV-2 genomic regions	Sensitivity	Reference
Charité, Berlin	E	3-5 copies/reaction	(30)
US CDC	N1 and N2	1-10 copies/reaction	<https://www.cdc.gov/coronavirus/2019-ncov/index.html> <https://www.fda.gov/media/134922/download>
Abbott	N and RdRP^*^	100 copies/mL	Package Insert
Roche	ORF1a and E	ORF1a=0.007 TCID50/mL	Package Insert
		E=0.004 TCID50/mL	
Biomanguinhos	E	50 copies/reaction	Package Insert
SeeGene	E, N, and RdRP	100 copies/reaction	Package Insert
Altona	E and S	0.1 PFUs/mL	Package Insert
Cepheid	E and N	250 copies/mL	Package Insert
Microbiomed	ORF3 and N	ORF 3=1.800 copies/μL	Package Insert
		N=4.239 copies μL	

^*^The two probes have the same fluorophore (FAM), thus the signal from both is summed. CDC: Centers for Disease Control and Prevention.

High-throughput labs able to process thousands of samples per day were and are still being created. In parallel, innovative approaches are in development and validation, in order to expand testing capacity, speeding up and simplifying the process. One approach has been to employ high-throughput sequencing (Next-Generation Sequencing - NGS) platforms able to read millions of bases simultaneously. Since reading of less than 100 bps with a low coverage is more than sufficient to assign a sample as SARS-CoV-2 detected or undetected, this strategy could solve the testing bottleneck. However, NGS does require RNA extraction and libraries are made of PCR amplicons. Thus, if one needs to extract RNA and further amplify it, in fact NGS in this context is being used as a sophisticated PCR reader. Moreover, NGS working flow still requires manual steps and takes 2-3 days to run one flow cell, contrasting to the automated flow of some PCR systems. These caveats indicate that NGS is more appropriate for epidemiological surveillance and phylogenetic studies but at the moment does not represent a practical solution for the routine high volume testing demand. Actually, countries with a large number of NGS platforms, such as China and the USA, rely exclusively on qPCR for the purpose of SARS-CoV-2 diagnosis.

Due to the RNA extraction constraint, researchers have shown that it is feasible to use the nasopharyngeal wash, in saline, water or viral transport medium, directly into the PCR tube, obviating the need for RNA extraction (34). As expected, there is some loss in sensitivity when comparing to extracted material, but hopefully new PCR mixtures that can surpass the inhibitory effect of unextracted substances will allow a huge increase in testing capacity.

Another alternative to the complexity of RT-qPCR and its reagents shortage is the loop-mediated isothermal amplification (RT-LAMP), which has shown a sensitivity similar to qPCR but faster and simpler. LAMP amplicons can be detected by visual inspection (35) or even by the collateral ssDNAse activity of the CRISPR-Cas12a system after target recognition, which adds a second level of specificity in addition to elevated sensitivity (36).

In spite of the viral RNA protocol adopted, it must be emphasized that the clinical sensitivity of molecular testing for COVID-19 has never achieved >99% as seen in many other infectious diseases. Both preanalytical and analytical conditions contribute to an average clinical sensitivity of 70% (37,38). So, it is not uncommon that nasopharyngeal samples from evident COVID-19 patients return as negative, while more invasive samples from the lower respiratory tract, such as broncoalveolar lavage from the same day, return positive for SARS-CoV-2 RNA (39).

### Serology

Serological assays represent the most common way for the diagnosis of viral infections. As early as March 2020, commercial kits became available in different formats: lateral flow immunoassays, enzyme-linked immunosorbent assays (ELISAs), and chemiluminescent immunoassays (CLIA). They also may differ in the antibody class detected (IgA, IgG, IgM, IgM/IgG, or total antibodies) and their composition of viral antigens.

However, due to the time between infection and antibody production, the so-called window period, detection of antibodies is not useful in the early symptomatic phase, when most patients seek clinical support. This limitation is magnified in the specific case of SARS-CoV-2 where the development of the classical marker of acute phase, IgM, does not seem to behave as usual. It may be an imperfection of the first generation of serological tests, but current IgM assays have failed to detect a significant fraction of truly infected (qPCR positive) subjects in the first 2 weeks from symptoms onset (40). Moreover, about 25% of the COVID-19 patients develop IgM concomitantly or after the appearance of IgG (41). Noticeably, in the third week after symptoms onset, the rate of seroconversion is >95%, allowing the use of antibody presence to estimate the exposure rate of a population, which is of key importance for the epidemiological monitoring of the epidemic but also to allow a planned safe return to professional and social life (42). Auspiciously, second generation assays are showing improved sensitivity (43) while the specificity has not been a significant drawback since the first-generation serological assays were introduced.

Today, it is premature to state that antibody-positive subjects are protected from a potential re-infection, which is likely because after millions of cases globally there is no well-documented report of re-infections (44,45). A related uncertainty is whether recovered patients with positive swabs are able to transmit the virus. In this sense, accumulating data are showing that the presence of neutralizing antibodies more than 8 days from symptom onset correlates inversely with the capacity of the isolated virus to infect susceptible cells *in vitro* (46).

## Clinical and therapeutic aspects

Clinical manifestation of COVID-19 can vary from mild and moderate to severe and critical disease, with patients requiring mechanical ventilation. Asymptomatic cases can occur, and asymptomatic and presymptomatic patients play an important role in the spread of the virus (47,48). The median incubation period is 4 days with more than 95% of symptomatic cases showing symptoms at 11 days (47,49). In 80% of persons, the disease is mild to moderate, presenting as a flu-like syndrome with resolution of symptoms in 10-14 days. The most common symptoms are fever, cough, myalgia, anorexia, and fatigue. Headache, chills, sore throat, anosmia, and ageusia are frequently described in different series. Uncommon presentations with different manifestations like diarrhea, rash, or abdominal pain can occur (49-51, Table 2). The Kawasaki syndrome has been described recently in children and adolescents (52). Twenty percent of patients present severe symptoms requiring hospitalization and about 5% will evolve to critical illness and intensive care unit (ICU) admission, the majority of them with acute respiratory distress syndrome (ARDS), requiring mechanical ventilation. Renal failure occurs in 30% of critically ill patients, most of them in need of hemodialysis (53,54). Overall case fatality rate varies between 1–6% but in patients who need mechanical ventilation, mortality can be as high as 61% (49,53). Median time for hospital admission is 7 days and for ICU admission, 9.5 days (8,53,54). Risk factors for severe disease and death are described in Table 3(50,51,55).

**Table 2:** Most described symptoms in COVID-19.

Most common symptoms	Less common symptoms
Fever ≥37.8°C	Anorexia
Cough	Sputum production
Dyspnea	Odynophagia
Myalgia	Confusion and dizziness
Fatigue	Headache
	Diarrhea
	Nausea/vomiting
	Abdominal pain
	Conjunctival congestion
	Anosmia and ageusia

**Table 3:** Risk factors for severe disease and death in COVID-19.

Risk factors
Older age (>65 years)
Chronic lung disease
Diabetes
Obesity (BMI ≥30)
Hypertension
Cardiovascular disease
Renal disease (end stage)
Liver disease

BMI: body mass index.

Severe COVID-19, with pneumonia and ARDS, is related to hyper-inflammation and release of pro-inflammatory cytokines. Pulmonary inflammation leads to rapid deterioration of pulmonary function and organ failure. Macrophage activation syndrome (MAS), known as cytokine storm, occurs specifically in lungs, with release of tumor necrosis factor (TNF)-α, interleukin (IL)-6, IL-1, and IL-8. Besides that, SARS-CoV-2 causes massive alveolar cell death that contributes to more inflammation. This process is reflected by elevated C-reactive protein (CRP), hyperferritinemia, increased level of IL-6 in blood, and altered liver function tests with coagulopathy. D-dimer and fibrinogen elevation can be observed in severe patients with COVID-19, due to extensive pulmonary microthrombosis. Lymphopenia occurs as result of induced immunosuppression (56,57).

For patient stratification, it is important to look for inferior respiratory symptoms. Those patients with a respiratory rate >22 breaths/minute or oxygen saturation (SatO_2_) <92% are considered at risk for complication and must be hospitalized. Chest computed tomography (CT) scan and calculation of the quick sepsis-related organ failure assessment (qSOFA) score is recommended for risk stratification. Patients with qSOFA=1, SatO_2_<92% in room air and extensive ground-glass opacities in >25% of lungs must be hospitalized in the medical ward. Criteria for ICU admission is qSOFA≥2 or qSOFA=1 plus SatO_2_≤92%, mechanical ventilation, arterial hypotension, persistent respiratory rate >30, or unconsciousness. One study proposed the use of lactic dehydrogenase (LDH), lymphocyte count, and CRP with a clinical route as a predictor score for mortality (58,59). Presence of lymphopenia, elevated LDH, hyperferritinemia, or altered D-dimer are predictors of severe disease and mortality.

As a new disease emerges, several drugs are proposed for treatment based on *in vitro* effectiveness against the etiological agent. However, their *in vivo* activity must be confirmed in randomized clinical trials. There are several drugs being tested. Chloroquine and hydroxychloroquine associated or not with azithromycin were first indicated as candidate drugs, although there are no randomized clinical trials supporting this indication. Only small uncontrolled trials were published, some of them show no benefit in reducing progression of the disease or mortality. Recently, studies published as preprints that showed some benefits were withdrawn by authors or after peer-review. Concerns with toxicity have arisen with studies showing increase in mortality due to cardiovascular adverse events with the use of chloroquine or hydroxychloroquine alone or in combination with a macrolide (60,61). Until randomized clinical trials show benefits of these drugs in the treatment of COVID-19, they should not be recommended.

Remdesivir, an RNA polymerase inhibitor and experimental drug from the beginning of this pandemic, is now allowed in the USA and Europe for treatment of hospitalized patients with COVID-19 after a randomized clinical trial comparing the drug with placebo showed shortened recovery time in the remdesivir group (11 *vs* 15 days in placebo arm) at preliminary analysis (62). The trial continues, now comparing remdesivir with remdesivir plus baricitinib, an anti-inflammatory drug that inhibits cytokines.

Tocilizumab, an anti-human IL-6 receptor monoclonal antibody, is being studied in severe and critical COVID-19 patients. One observational study in 21 patients, 20 with ventilatory support, showed reduction in respiratory support in 75% of patients. Randomized controlled studies are needed to recommend the drug, although it may be beneficial in the treatment of patients with cytokine storm (63).

Corticosteroid therapy was associated to increased mortality in patients with severe or critical COVID-19, but the studies were not randomized and involved heterogeneous populations. One study in patients with ARDS showed a decrease in the risk of death with the use of methylprednisolone (64). More controlled studies are needed to recommend this drug as adjuvant treatment in patients with severe disease.

## Conclusions and future perspectives

There are two interrelated variables that are key to project the future of the pandemic: immunity and viral evolution. As previously stated, >95% of the exposed subjects do develop a detectable IgG. What is unknown is whether this signifies sterilizing immunity and, if it does, for how long. There is some evidence that re-infection is unlikely, at least in the short term (65). However, we still need more time, data, and experience to prove that the presence of IgG is indeed a correlate of immunity for SARS-CoV-2. Even if antibodies wane, protection may remain through cellular memory and fast regain of antibodies production upon exposure, as observed in other viral infections. In this scenario, seroprevalence rates will indicate the actual risk for a determined population, and above a level of infection, estimated as 60-70%, the herd immunity may ensue (66). Viral evolution correlates with immunity in the context of viral escape. It is recognized that the generation of a large progeny composed of minor genomic variants, the quasispecies concept (67), is a phenomenon common to all RNA viruses. In contrast, SARS-CoV-2 has shown moderate substitution rates that may be attributed to the proofreading activity of its RNA polymerase (68), lacking on most RNA viruses. Thus, the optimistic scenario is one where immune response to SARS-CoV-2 is long lasting and, in populations with high seroprevalence, new cases will be rare and self-contained, while the pessimistic one is where immunity lasts for only a few months, as seen for other human coronaviruses (69), and thus we will continue to witness periodic outbreaks, as the majority of the population remains susceptible.

Countries and regions are currently at different stages of the epidemic. During these 6 months, it became more evident which approaches work better, when saved lives and the preservation of the health system are the parameters considered. Social isolation is extremely efficient in flattening the curve of the disease but it is unsustainable in the long run. Identifying, by massive molecular testing, asymptomatic and presypmtomatic carriers that are contacts of index symptomatic SARS-CoV-2 RNA positive cases, is clearly the strategy that reduces the rate of new infections while allowing the relaxation of containment measures (70).

Meanwhile, there are great expectations for a definite solution represented by an effective vaccine. There are currently many candidates including a few in phase 3 trials (71). Until we have a vaccine, it is likely that SARS-CoV-2 will continue to cause outbreaks requiring new rounds of social closure and protection to susceptible groups. Masks will be mandatory in public spaces and events. Hopefully, this tragedy will enhance international cooperation, empower multinational organizations, and highlight to the world the importance of science.
